# Color Shade Nets Affect Plant Growth and Seasonal Leaf Quality of *Camellia sinensis* Grown in Mississippi, the United States

**DOI:** 10.3389/fnut.2022.786421

**Published:** 2022-02-02

**Authors:** Qianwen Zhang, Guihong Bi, Tongyin Li, Qiushuang Wang, Zhiheng Xing, Judson LeCompte, Richard L. Harkess

**Affiliations:** ^1^Department of Plant and Soil Sciences, College of Agriculture and Life Sciences, Mississippi State University, Mississippi State, MS, United States; ^2^Key Laboratory of Tea Plant Resources Innovation and Utilization, Tea Research Institute, Guangdong Academy of Agricultural Sciences, Guangzhou, China

**Keywords:** tea, cold damage, polyphenols, carbohydrates, caffeine, catechins, protected culture

## Abstract

Shading modifies the microenvironment and can provide plants with some protection from frequent heat, drought, frost, and hail induced by climate change and has the potential to improve plant growth, yield, and quality. Tea (*Camellia sinensis*) is an ancient plant originating from tropical and subtropical regions and prefers to grow in partial shade under the forest canopy. The emerging tea industry in the United States (US) requires research support on establishing tea fields in novel environmental conditions as well as on producing high-quality tea products. This study investigated the effects of black, blue, and red shade nets on tea plant growth and seasonal leaf qualities in the southeastern US with a humid subtropical climate. When compared to no-shade control, black, blue, and red shade nets increased plant growth index (PGI), net photosynthetic rate (*P*_n_), and stomatal conductance (*g*_s_), decreased air and leaf surface temperatures in summer, and reduced cold damage in winter. No significant difference was found among the black, blue, and red shade nets on tea plant growth. Varying contents of total polyphenols, carbohydrates, free amino acids, L-theanine, gallic acid, caffeine, and catechins in fresh tea leaves were observed among different shade treatments and harvesting seasons. 69.58% of the variations were depicted in a biplot by principal component analysis. Red shade was considered helpful for improving green tea quality by increasing the content of L-theanine and free amino acids in tea leaves collected in spring and fall when compared to no-shade control.

## Introduction

Climate change, including increasing temperatures, intense heat waves, frequent drought, frost, heavy rain, flooding, and hail, is becoming one of the biggest challenges in agricultural production (1–3). Alternative production practices such as shading can help to alleviate some of the negative impacts of climate change. Shading affects radiant exposure, temperature, humidity, soil conditions, and microorganism biodiversity and can protect plants from sunburn, frost, and hail (3–8). Shading was reported to improve the yield and quality of a number of horticultural crops, including fruits, vegetables, ornamentals, herbs, and tea (9–15). The application of shade on horticultural crops is becoming more popular in the recent years (16).

*Camellia sinensis* (*C. sinensis*) is the botanical source of various tea products for thousands of years (17). Tea is an ancient plant originated from southwestern China about 5,000 years ago (18). It is a broad-leaved perennial evergreen tree or shrub, adapted to tropical to subtropical climates. Tea grows vigorously in temperatures between 18 and 30°C, with the ability to withstand temperatures from−16 to 40°C and annual rainfall of 1,250 to 6,000 mm (19). The first scientific work of the world on tea the book “Cha Jing,” also known as “The Tea Classic” or “The Classic of Tea” described that tea plants grow the best in the forest, on the sunny hillside, with natural shading from tree canopy (20). It reveals that tea plants prefer diffused light. While for tea fields with excessive sun exposure, shading is a traditional method to enhance the plant growth. The history of shading application on tea plants dates back to the mid-1,800s (4, 21, 22).

Tea is the most popular beverage, second only to water, with world total production of 6.49 million tons harvested from over 5 million ha across 47 countries in 2019 (23). Leading tea producing countries including China, India, and Kenya produce over 70% of the total world tea (18, 23). The United States is the third largest tea importer in the world with Americans consuming over 14 billion liters of tea in 2020 (24). With increasing demand for domestically grown tea, tea production has attracted interests from growers across the US (25). Mississippi is one of the state's leading the effort in tea production. Mississippi is located in a humid subtropical area, with average annual temperatures from 17°C in the north to 20°C along the Gulf Coast and annual precipitation ranging from 1,270 to 1,651 mm across the state (26). The United States Department of Agriculture (USDA) hardiness zone is a guide on which plant can thrive at a location based on average annual minimum temperatures. Tea can be grown in the USDA plant hardiness zones 6–9 (27), while Mississippi is in 7–9. Thus, the climate in Mississippi is suitable for growing tea, which has been proven by the establishment of multiple small scale commercial tea farms in the state (25). However, challenges for tea growers in Mississippi include heat stress with summer temperatures exceeding 32°C for over 100 days annually and cold damage caused by winter freezes and spring frosts. Shade nets can potentially alleviate some of these stresses on tea plants by modifying the microenvironment and providing physical protection.

With more researchers contributing to the practical implementation of shade nets in agriculture, more sophisticated studies on shade nets have been conducted, including the spectral selective (color) shade nets (28). Light is one of the most important factors affecting plant growth. Conventional shade nets were used to reduce light intensity, while color shade nets manipulate spectral bands of light (8). Spectral bands received by plants can affect crop quality, yield, and biosynthesis of metabolites. Different colors of shade nets reflect or absorb different wavelengths of light. Blue shade nets absorb ultraviolet, red, and far-red light spectral bands, enriching the blue light spectra, while red shade nets absorb ultraviolet, blue, and green light spectral bands, enriching red and far-red light spectra (16). Black shade nets do not modify the spectral composition of light received by plants (29). Most shade studies on tea plants focused on black shade nets, which reduce light intensity and were mainly used to cover tea plants for a short period of time in summer and fall (9, 11, 30–33). Little to no information is available regarding effects of different colored shade nets on tea plant growth and leaf quality when shade nets were used year-round. This study is one of the few recent research efforts to investigate the effects of black, blue, and red shade nets on tea plants grown in the southeastern United States. The objective of this study was to investigate the effects of black, blue, and red shade nets on plant growth and seasonal leaf quality of *C. sinensis* to better understand the effectiveness of this technique to enhance tea productivity in Mississippi, US.

## Materials and Methods

### Plant Cultivation and Experimental Design

A total of 1-year-old tea plants (*C. sinensis* var. *sinensis*) of “small leaf” cultivar vegetatively propagated from cuttings and grown in one-gallon containers were transplanted into a field located at the R. R. Foil Plant Science Research Center at Mississippi State University (latitude 33°29′N and longitude 88°47′W) in Starkville, Mississippi, US, in May 2016. The field soil was a Stough fine sandy loam with a pH of 4.9. Plants were planted in three double row hedges, with 0.76 m between plants within a row, 0.91 m between inner rows, and 1.83 m between double rows. Plants were pruned to a uniform height of 25 cm after transplanting and fertilized with Osmocote® Plus 15-9-12, 8–9 months standard pattern release fertilizer (15N-3.9P-10K, ICL Specialty Fertilizers, Summerville, South Carolina, USA) at a medium rate of 112 g m^−2^. Plants were irrigated as needed through drip irrigation. Pine bark mulch was used between rows for weed control. Local monthly air temperature, accumulated precipitation, and relative humidity data within the research duration from January 2017 to March 2019 were obtained from the USDA Natural Resources Conservation Service website.

Shade nets were installed after transplanting in the field. Four shade treatments including black, red, and blue shade nets providing 50% shade (ChromatiNet® Polysack Plastic Industries Ltd., Nir Yitzhak, Israel), along with an unshaded control. Each shade net was 3.66 m wide and 8.53 m long, suspended above the tea plants by 4 metal fence posts. The experiment was a completely randomized block design with three replications and shade treatment as the experimental factor. There were 20 plants (subsamples) for each shade treatment within each replication.

### Plant Performance

Plant growth index (PGI), cold tolerance, leaf surface temperature, and photosynthetic activities were evaluated. PGI and cold tolerance of each plant under each treatment were measured in February 2018 and 2019. PGI was calculated as the average of plant height, width 1 and width 2, where plant width 1 was the greatest width of a plant and width 2 was the perpendicular width to width 1. Cold tolerance of each plant was determined by the percentage of foliage showing cold-damaged symptoms as described by Luo (19). Three plants from each experimental unit were randomly selected and measured for photosynthetic characteristics. Photosynthetically active radiation (*PAR*), net photosynthetic rate (*P*_n_), stomatal conductance (*g*_*s*_), leaf transpiration rate (Trmmol), air temperature, and leaf temperature were measured between 1,000 and 1,300_HR_ on September 19, 2018 using a portable photosynthesis system (Li-6400 XT; LI-COR Biosciences, Lincoln, Nebraska, USA). One of the newest fully expanded leaves, not shaded by any other leaves or branches, was enclosed into a 2-cm^2^ leaf chamber for measurement. When photosynthetic data were collected, the weather was sunny without overcast. Block temperature was maintained at 34–36°C according to the real-time ambient air temperature. Reference carbon dioxide (CO_2_) was set at 400 μmol m^−2^ s^−1^. Relative humidity (RH) was adjusted to be 40 to 60%. *PAR* in the leaf chamber was set to be 1,600 and 750 μmol m^−2^ s^−1^ for no-shade control and shade treatments, respectively. The fluorometer was used to be the light source. The photosynthesis system was warmed up and prepared according to the instructions of the manufacturer; then, it was brought to the field for measurement approximately 1 h after being started.

### Chemical Components and Bioactive Metabolites Analyses in Fresh Tea Leaves

Fresh tea leaf samples containing one terminal bud and two leaves were collected in spring, summer, and fall in 2018 (on 10 April, 12 July, and 18 October, respectively). Each sample was oven-dried at 120°C for 5 min (for enzymatic deactivation) and then 60°C to a constant weight. Dried samples were ground to pass a 40-mesh (0.42 mm) sieve using a Thomas Wiley Mini-Mill (Thomas Scientific, Waltham, Massachusetts, USA). For each experimental unit, three tea infusions were prepared for the measurement of carbohydrates, free amino acids (AAs), total polyphenols (TPs), L-theanine, catechins, and caffeine. For each tea infusion, 0.6 g of ground leaf samples was weighed to make 100 ml tea infusion as described by Zhang et al. (25).

Carbohydrate content was determined by the anthrone-sulfuric acid method (34) with results being expressed as dextrose equivalents in % dry matter. Total polyphenol was analyzed using the Folin-Ciocalteu method (35) with results being expressed as gallic acid equivalents (GAEs) in % dry matter. Content of free AAs was measured by the ninhydrin dyeing method and expressed as glutamic acid equivalents in % dry matter (36). Solution absorbance in the test of carbohydrates, TP, and free AAs content was measured using a spectrophotometer (Nicolet Evolution 100, Thermo Fisher Scientific, Waltham, Massachusetts, USA). L-theanine, caffeine, and catechins in tea infusion were determined by the high-performance liquid chromatography (HPLC) method, performed on the 1260 Infinity II LC System (Agilent Technologies, Wilmington, Delaware, USA) with the G1315C Diode Array Detector (Agilent Technologies, Wilmington, Delaware, USA), using a TC-C18 column (4.6 mm × 250 mm, 5 μm; Agilent Technologies, Wilmington, Delaware, USA) with a TC-C18 guard column (4.6 mm × 12.5 mm; Agilent Technologies, Wilmington, Delaware, USA). The analyses of caffeine and catechins followed the protocol described by Zhang et al. (25). The content of L-theanine in tea infusions was determined using the HPLC method described by Li et al. (37).

L-theanine, gallic acid (GA), caffeine, catechins, and Folin-Ciocalteu reagent were purchased from Sigma-Aldrich (St Louis, Mosby, USA). References and mobile phase used in HPLC were HPLC grade. All the other chemicals were analytical grade and were purchased from Thermo Fisher Scientific (Waltham, Massachusetts, USA).

### Statistical Analyses

Statistical analyses were performed in Statistical Analysis System (SAS) (version 9.4; SAS Institute, Cary, North Carolina, USA). Results in figures and tables were expressed as mean ± SEM. The effect of treatments was evaluated by the ANOVA followed by Tukey's honestly significant difference (HSD) test at *p* ≤ 0.05 for mean separations. A logarithmic transformation was made on cold tolerance data (%) to meet the assumption of normality. Chemical compositions data collected from three seasons under four treatments were further analyzed using principal component analysis (PCA) in XLSTAT (version 2021; Addinsoft, Paris, France).

## Results

### Local Weather Conditions

**Figure 1:**
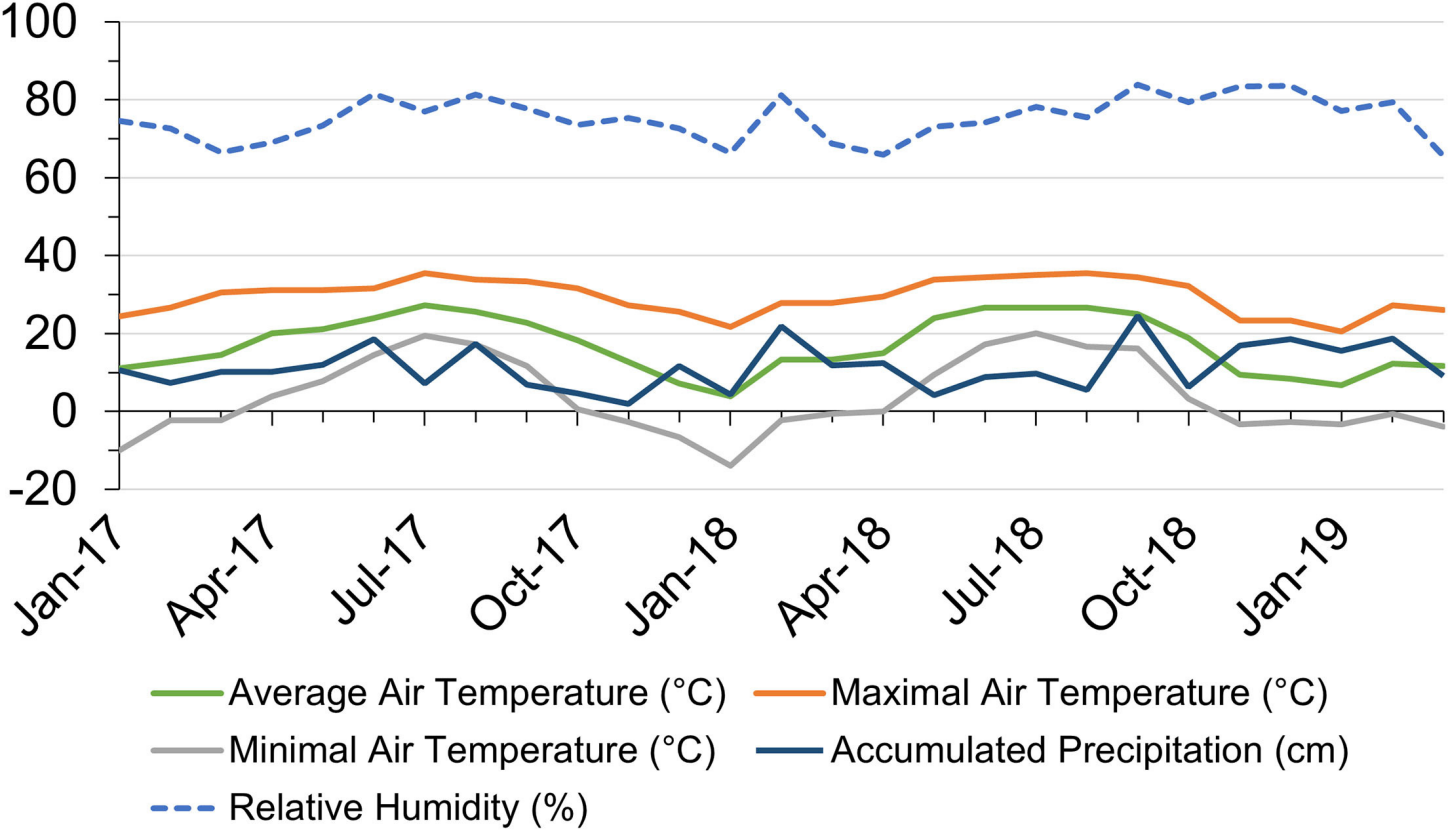
Local monthly air temperature and accumulated precipitation in Starkville, Mississippi (MS), United States within the research duration from January 2017 to March 2019. Data source: https://wcc.sc.egov.usda.gov/reportGenerator/.

Monthly air temperature, precipitation, and RH from January 2017 to March 2019 in Starkville, Mississippi, USA are shown in Figure 1. Average air temperature ranged from 3.9°C in January 2018 to 27.2°C in July 2017. The highest temperature reached 35.6°C in July 2017 and the lowest temperature dropped to−13.9°C in January 2018. In 2017, there were 8 months with the highest temperature reaching above 30°C, while in 2018, there were 6 months with daily maximal temperatures above 30°C. Monthly accumulated precipitation ranged from 1.9 cm in November 2017 to 24.6 cm in September 2018. Heavy precipitation randomly occurred throughout the experiment duration. Monthly average RH shared similar trends with precipitation, ranging from 65.6% in March 2019 to 83.9% in September 2018.

### Plant Growth Indices

**Figure 2:**
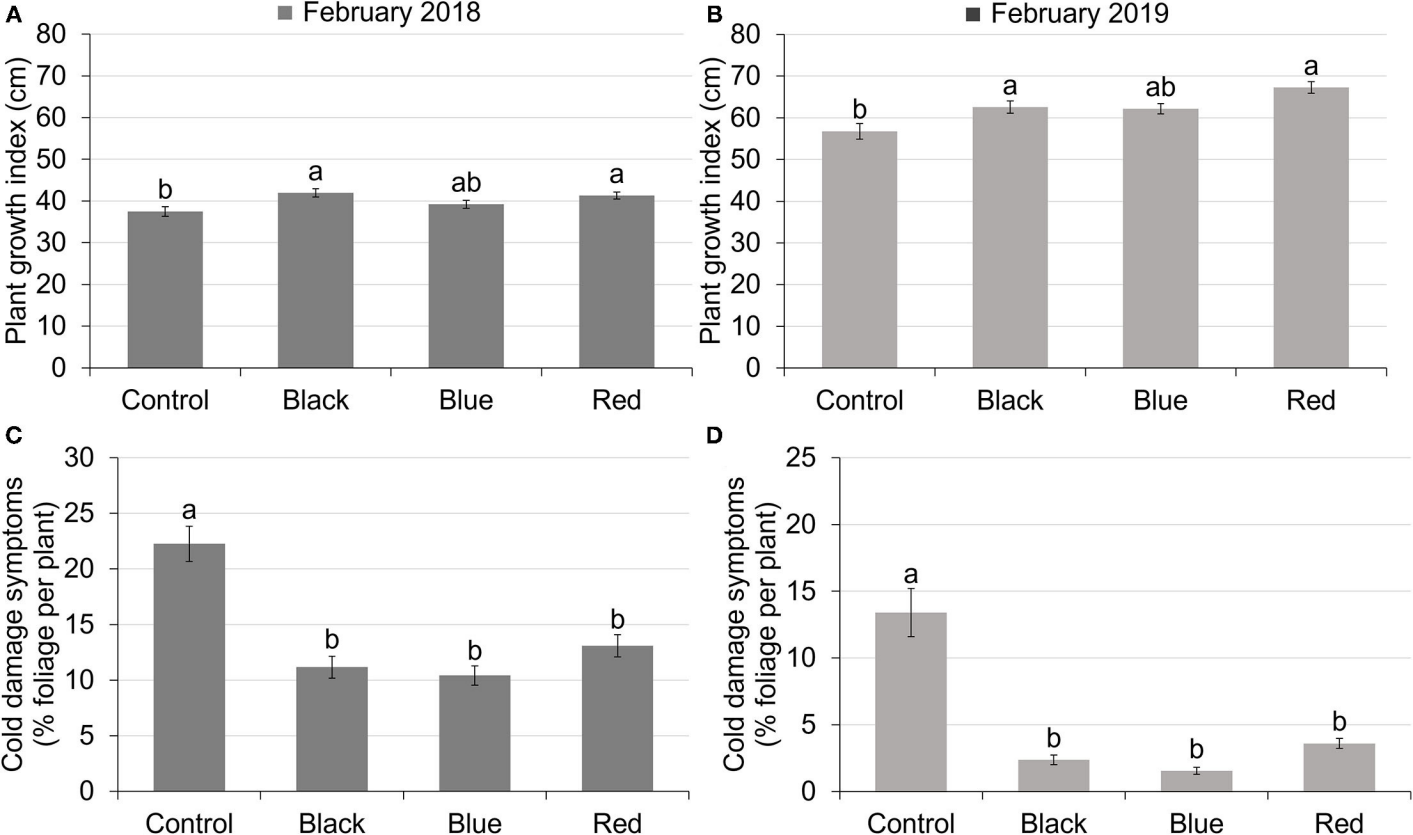
Plant growth index of tea plants in February 2018 **(A)** and February 2019 **(B)** under different treatments. Plant growth index was calculated as the average of plant height, width 1 (widest points apart) and width 2 (perpendicular to width 1). The percentage of foliage showing cold-damaged symptoms of tea plants in February 2018 **(C)** and February 2019 **(D)** under different treatments. Different letters on top of the columns within each figure represent significant difference between the treatments indicated by Tukey's honestly significant difference (HSD) test at *p* ≤ 0.05.

Use of shade nets affected plant growth indices. In both 2018 and 2019, plants grown under black and red shades have similar PGIs as plants grown under blue shade, but higher PGIs compared to those grown under no shade. Blue shade resulted in similar PGI to no shade (Figures 2A,B). In 2018, PGIs ranged from 37.50 to 41.92 (Figure 2A). In 2019, PGIs ranged from 56.76 to 67.28 (Figure 2B). PGIs were higher in 2019 than in 2018, with an average increase of 55.5%.

### Cold Damage

All the shades reduced plant cold damage compared to no-shade control in both 2018 and 2019 (Figures 2C,D). The percentage of foliage showing cold damage symptoms for plants grown under shade nets ranged from 10.42 to 13.09% in February 2018 and 1.54 to 3.60% in February 2019 compared to 22.25% in 2018 and 13.41% in 2019 for plants grown with no shade. Cold damage was more severe in February 2018 than February 2019 for all the treatments due to low temperatures in January 2018 (Figure 1).

### Photosynthetic Characteristics

Shade nets affected various photosynthetic characteristics of tea plants when measured in September 2018 (Table 1). Compared to no-shade control, black, blue, and red shades significantly increased *P*_n_ and *g*_s_, but decreased *PAR*, air temperature, and leaf temperature. *P*_n_ under shade nets was 40.14 to 45.75% higher than control. Although *g*_s_ of tea plants grown under shades was 36.36 to 54.55% greater than no-shade control, there was no difference in leaf transpiration rate (Trmmol) among all the treatments. Shade nets resulted in a *PAR* range of 530.08 to 723.75 μmol m^−2^ s^−1^, which was 54.70 to 66.82% of full sunlight (1597.73 μmol m^−2^ s^−1^) as measured in control. Shades decreased air and leaf temperatures by 2.08 to 2.19°C and 3.08 to 3.79°C, respectively. However, *P*_n_, *g*_s_, *PAR*, and leaf temperature did not differ among black, blue, and red shades.

**Table 1:** Photosynthetic characteristics of tea plants under different color shade treatments.

**Treatment**	***P*_n_** ** (μmol CO_**2**_ m^**−2**^ s^**−1**^)**	***g*_s_** ** (mol H_**2**_O m^**−2**^ s^**−1**^)**	**Trmmol** ** (mmol H_**2**_O m^**−2**^ s^**−1**^)**	***PAR*** ** (μmol m^**−2**^ s^**−1**^)**	**Air temperature** ** (**°**C)**	**Leaf temperature** ** (**°**C)**
Control	7.30 ± 0.57 b	0.11 ± 0.01 b	4.13 ± 0.21 a	1597.73 ± 26.53 a	36.78 ± 0.03 a	38.47 ± 0.33 a
Black	10.23 ± 0.45 a	0.15 ± 0.01 a	3.88 ± 0.21 a	723.75 ± 64.06 b	34.70 ± 0.03 b	35.39 ± 0.11 b
Blue	10.64 ± 1.03 a	0.17 ± 0.02 a	4.47 ± 0.38 a	530.08 ± 24.51 b	34.59 ± 0.05 c	34.68 ± 0.27 b
Red	10.43 ± 0.78 a	0.17 ± 0.01 a	4.22 ± 0.24 a	709.65 ± 82.66 b	34.69 ± 0.02 b	35.13 ± 0.14 b
*P*-value	0.0004	0.0029	0.3562	<0.0001	<0.0001	<0.0001

*P_n_, net photosynthetic rate; g_s_, stomatal conductance; Trmmol, leaf transpiration rate; PAR, photosynthetically active radiation under shade nets or no-shade control*.

*Means followed by different letters within each column suggest significant difference between treatments according to Tukey's honestly significant difference (HSD) test at p < 0.05*.

### Total Polyphenols

**Figure 3:**
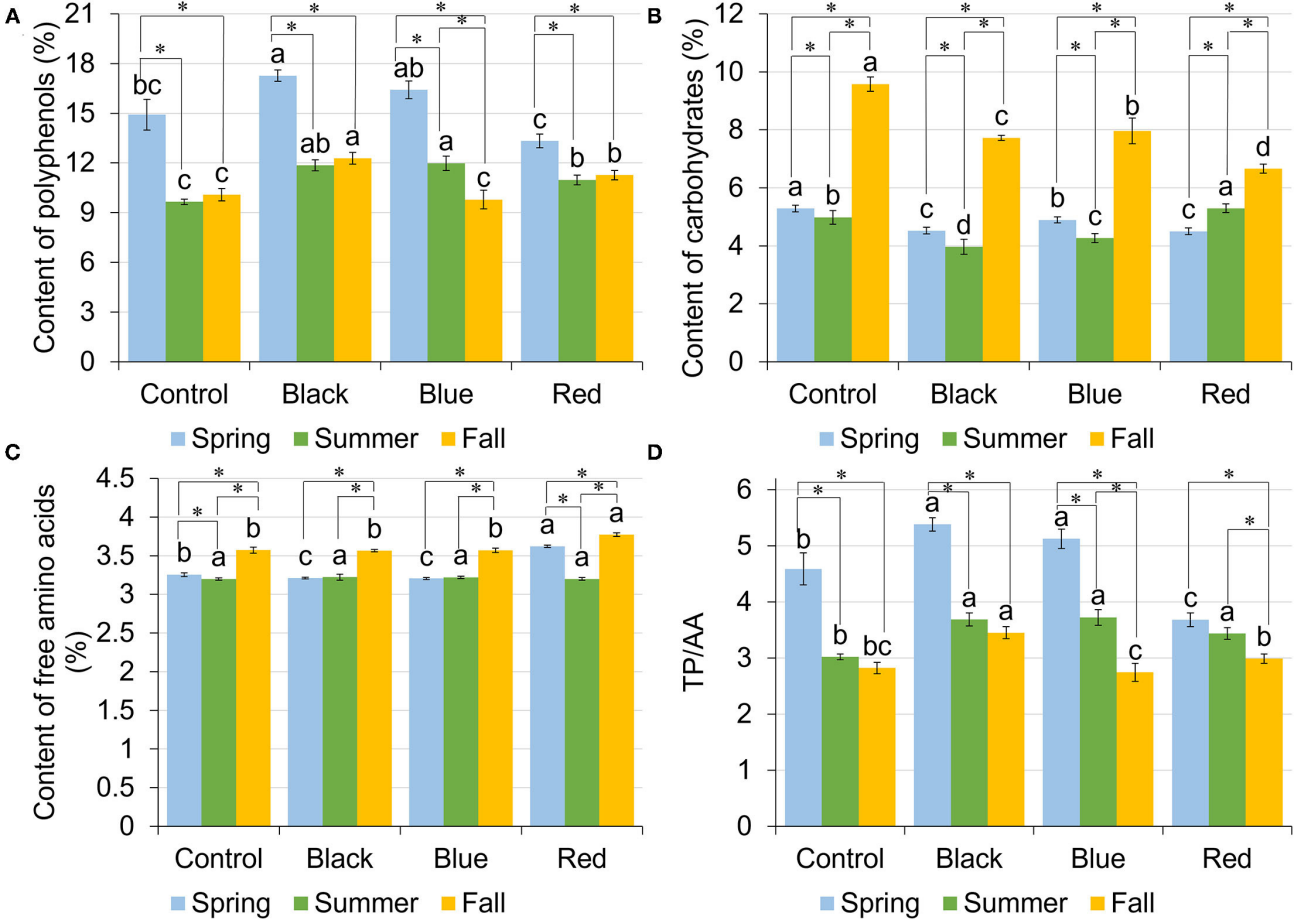
Content of total polyphenols (TPs) [gallic acid equivalents (GAE) % dry matter] **(A)**, carbohydrates (dextrose equivalents % dry matter) **(B)**, free amino acids (AAs) (L-theanine equivalents % dry matter) **(C)**, and the ratio between TPs and free AAs **(D)** in tea leaves collected from plants under different shade treatments in spring, summer, and fall. Different letters on top of the same-colored columns represent significant difference between the shade treatments within one season; *connected different columns represent significant difference within one shade treatment between seasons. All the significant differences were indicated by Tukey's HSD test at *p* ≤ 0.05.

Total polyphenols (TP) content in tea leaf varied among shade treatments and harvesting seasons (Figure 3A). In spring, plants grown under black and blue shades resulted in the highest TP content of 17.27 and 16.41%, respectively, followed by no-shade control (14.92%) and red shade (13.32%). In summer, all the shades had higher TP content compared to no-shade control. In fall, the highest TP was found under black shade net (12.29%), followed by red shade of 11.27%, with control and blue shade having the lowest TP of 10.08 and 9.79%, respectively. Across three harvesting seasons, TP was significantly higher in spring than summer or fall. Under black and red shades and no-shade control, tea leaf samples had comparable TP content in summer and fall within each treatment. For blue shade, TP was higher in summer than in fall.

### Carbohydrates

Carbohydrate content ranged from 4.50 to 5.29%, 3.97 to 5.29%, and 6.66 to 9.57% in spring, summer, and fall, respectively (Figure 3B). In spring and fall, no-shade control resulted in the highest content of carbohydrates compared to other treatments. Blue shade resulted in the second highest carbohydrate content, with red shade having the lowest carbohydrate content. In summer, however, the highest carbohydrate content was found under red shade, followed by control and blue shade, with black shade having the lowest carbohydrate content. Within each treatment, control, black, and blue shared the same seasonal trend, where highest content was found in fall, followed by spring, with lowest content in summer. Red shade had the highest carbohydrate content in fall, but the carbohydrate content in spring was lower than in summer.

### Free Amino Acids

Among four shade treatments, the content of free amino acids (AA) ranged from 3.20 to 3.62%, 3.20 to 3.22%, and 3.56 to 3.77% in spring, summer, and fall, respectively (Figure 3C). In spring, the highest content of AA was found in tea plants grown under red shade. Control had the second highest AA content of 3.25%, with black and blue having the lowest AA content. In summer, there was no significant difference in the AA content among shade treatments. In fall, red shade had the highest AA content, with no difference among the other three treatments. Within each treatment, control and red shade shared a similar seasonal trend, where the highest AA content was found in fall and the lowest AA content was found in summer. Under black and blue shades, tea leaf samples had the highest AA content in fall, with no difference between spring and summer.

### L-Theanine

The content of L-theanine ranged from 0.51 to 0.86%, 0.46 to 0.90%, and 0.53 to 0.91% in spring, summer, and fall, respectively (Table 2). A representative HPLC chromatogram of L-theanine in tea leaf sample collected under red shade in fall is shown in Supplementary Figure 1. There was no significant difference in the L-theanine content across three harvest seasons within each shade treatment, except that blue shade resulted in the higher L-theanine content in fall than summer. Compared to no-shade control, black, blue, and red shades increased the L-theanine content in tea leaves within each season. Generally, the highest L-theanine content was found in tea plants grown under red shade.

**Table 2:** Content of L-theanine in tea in tea leaves collected from plants under different shade nets in spring, summer, and fall.

	**L-theanine (% dry matter basis)**			
**Treatment**	**Spring**	**Summer**	**Fall**	***P*-value**
Control	0.51 ± 0.03 Ca	0.46 ± 0.05 Ca	0.53 ± 0.04 Ba	0.4819
Black	0.67 ± 0.05 Ba	0.74 ± 0.03 ABa	0.83 ± 0.06 Aa	0.0767
Blue	0.71 ± 0.04 ABab	0.65 ± 0.06 Bb	0.80 ± 0.01 Aa	0.0458
Red	0.86 ± 0.04 Aa	0.90 ± 0.05 Aa	0.91 ± 0.05 Aa	0.6867
*P*-value	<0.0001	<0.0001	<0.0001	

*Means followed by different uppercase letters within each column suggest significant difference between shade treatments and different lowercase letters within each row suggest significant difference between harvesting seasons according to Tukey's HSD test at p < 0.05*.

### Total Polyphenols/Free Amino Acids

Total polyphenols/free amino acids (TP/AA) varied among shade treatments and three harvest seasons (Figure 3D). In spring, tea plants grown under black and blue shades had the highest TP/AA of 5.38 and 5.12, respectively. Red shade had the lowest TP/AA of 3.68. In summer, shades had the higher TP/AA than no-shade control. In fall, TP/AA ranged from 2.74 to 3.45, with black shade having the highest TP/AA and blue shade having the lowest TP/AA. Under red shade, spring had the highest TP/AA of 3.68, followed by summer had the lowest TP/AA of 3.44, while fall had the lowest TP/AA of 2.99.

### Gallic Acid

**Figure 4:**
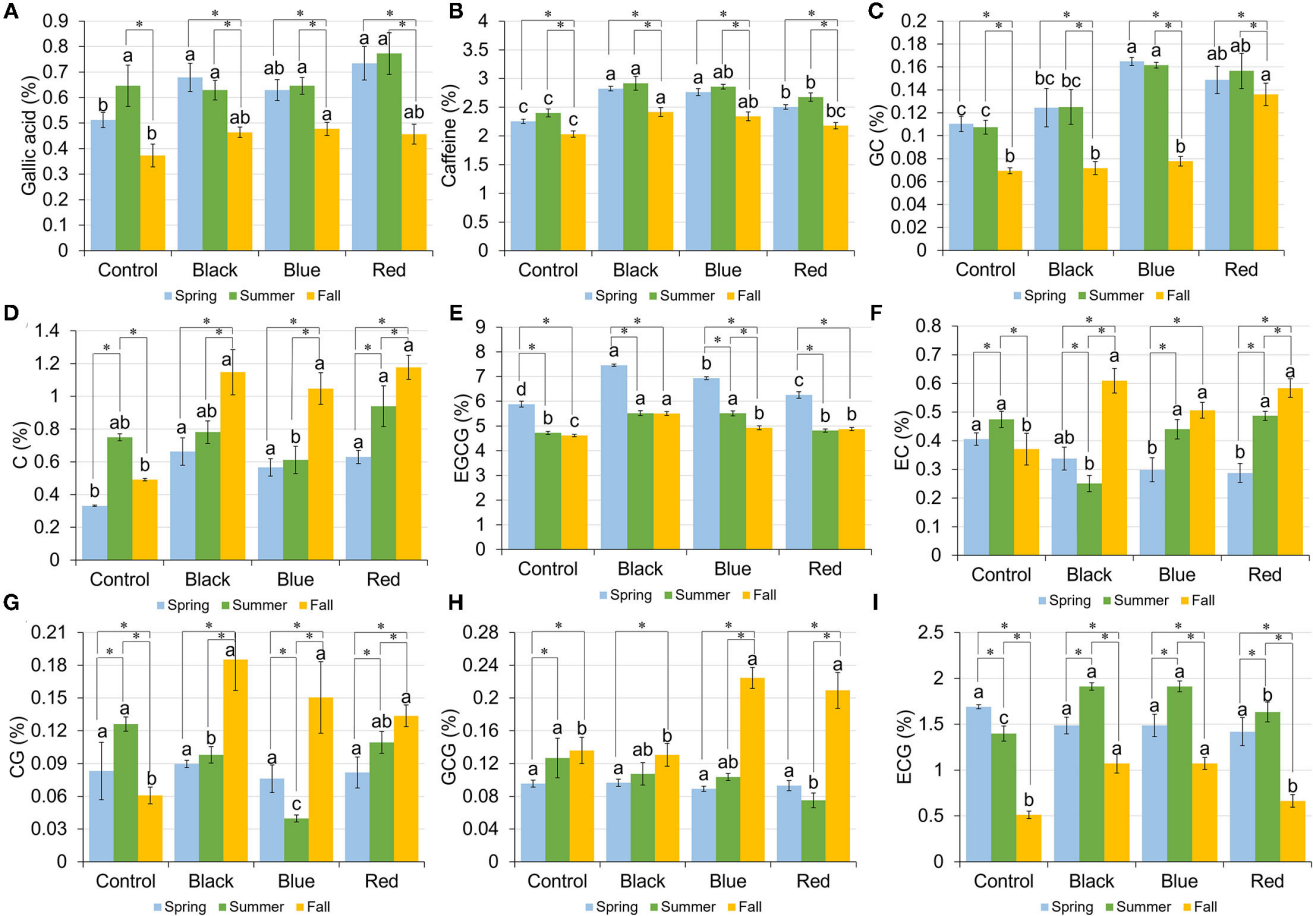
Content of gallic acid **(A)**, caffeine **(B)**, gallocatechin (GC) **(C)**, catechin (C) **(D)**, epigallocatechin-3-gallate (EGCG) **(E)**, epicatechin (EC) **(F)**, catechin-3-gallate (CG) **(G)**, gallocatechin-3-gallate (GCG) **(H)**, and epicatechin-3-gallate (ECG) **(I)** in tea leaves collected from plants under different shade treatments in spring, summer, and fall. Contents were presented as percent of dry matter basis. Different letters on top of the same-colored columns represent significant difference between the shade treatments within one season; *connected different columns represent significant difference within one shade treatment between seasons. All the significant differences were indicated by Tukey's HSD test at *p* ≤ 0.05.

The content of GA ranged from 0.51 to 0.73%, 0.63 to 0.77%, and 0.37 to 0.48% in spring, summer, and fall, respectively (Figure 4A). A representative HPLC chromatogram of GA, caffeine, and catechins in tea leaf sample collected under black shade in spring is shown in Supplementary Figure 2. In spring, plants grown under black and red shades had the higher GA content compared to no-shade control. In summer, all the treatments had the similar GA content. In fall, blue shade increased the GA content compared to control. Black, blue, and red shade shared a similar seasonal trend in the GA content, with spring and summer having the higher GA content than fall. In no-shade control, summer had the higher GA content than fall.

### Caffeine

The caffeine content ranged from 2.26 to 2.82%, 2.40 to 2.92%, and 2.03 to 2.42% in spring, summer, and fall, respectively (Figure 4B). In spring and summer, shades increased the caffeine content in tea leaves compared to no-shade control. In fall, black and blue shades resulted in the higher caffeine content than no-shade control. Within each shade treatment, tea leaves collected from different seasons had the similar caffeine content trend, with fall having the lowest caffeine content.

### Catechins

Catechins including gallocatechin (GC), catechin (C), epigallocatechin-3-gallate (EGCG), epicatechin (EC), catechin-3-gallate (CG), gallocatechin-3-gallate (GCG), and epicatechin-3-gallate (ECG) were measured using the HPLC method; their amounts in tea leaves under different shade treatments in spring, summer, and fall are shown in Figures 4C–I, respectively. In spring, three shades increased the contents of GC, C, and EGCG in tea leaves compared to control; blue and red shades resulted in the lower EC content than control; the contents of CG, GCG, and ECG did not vary significantly among four shade treatments. In summer, blue and red shades increased the GC content; black and blue shades increased the EGCG content, but decreased the CG content; black shade decreased the EC content; red shade decreased the content of GCG; all the shades increased the ECG content in tea leaves when compared with control. In fall, black, blue, and red shades increased the contents of C, EGCG, EC, and CG in tea leaves compared to control; red shade had the highest GC content compared to the other three treatments; blue and red shades resulted in the higher GCG content than black shade or control; black and blue shades increased the ECG content compared to red shade or control. Within each treatment, the trends of catechins among seasons varied. It was found that under black, blue, and red shades, the contents of C, EC, and CG in fall were higher than those in spring and summer.

### Principal Component Analysis

**Figure 5:**
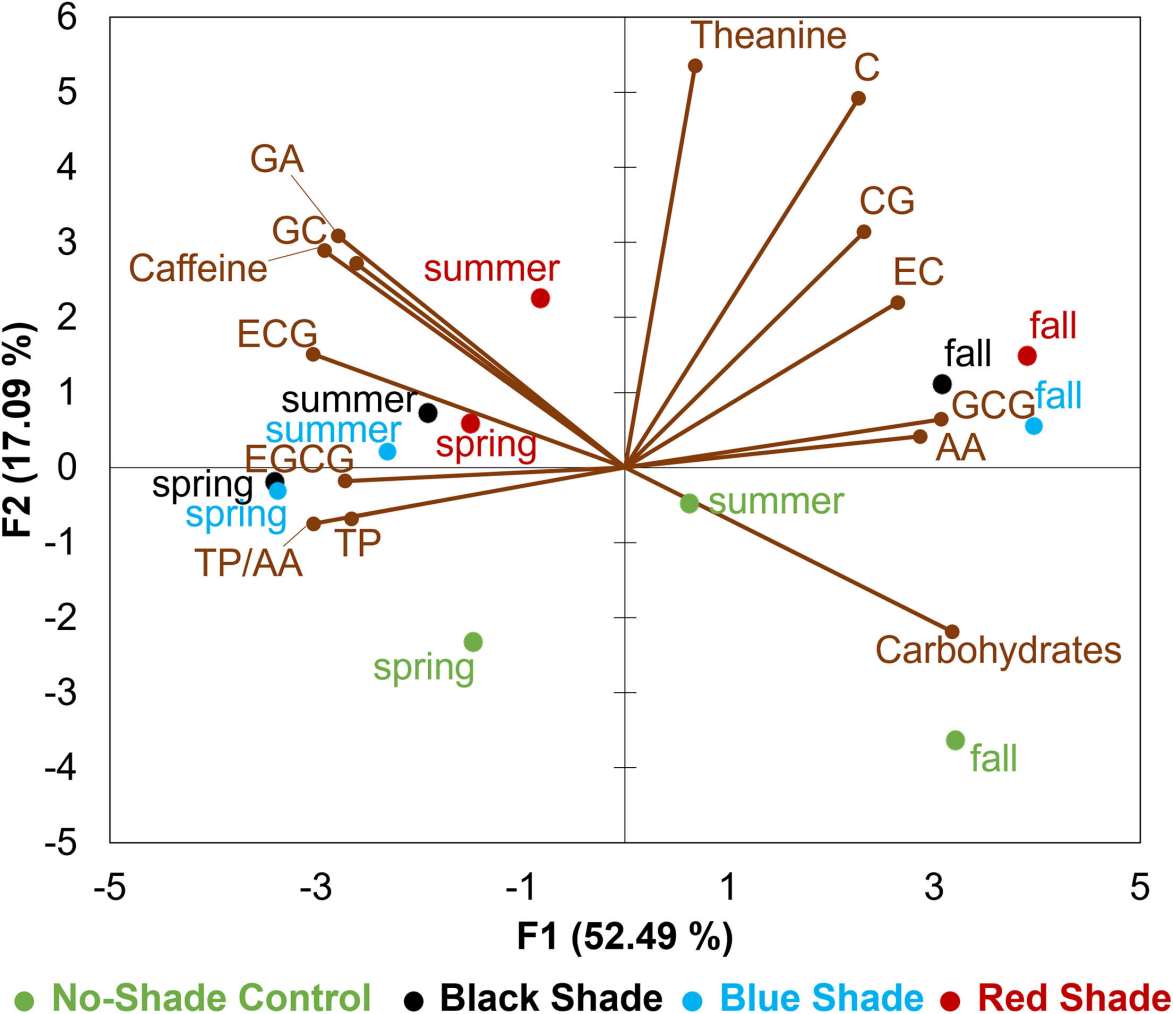
Principle component analysis (PCA) biplot for chemical compositions in tea leaves collected from plants under different shade treatments in spring, summer, and fall. GA stands for gallic acid, ECG stands for epicatechin-3-gallate, EC stands for epicatechin, GCG stands for gallocatechin-3-gallate, AA stands for free AAs, CG stands for catechin-3-gallate, C stands for catechin, EGCG stands for epigallocatechin-3-gallate, TP stands for TPs, and GC stands for gallocatechin.

Tea leaf samples were described by 14 chemical compositions including contents of TP, carbohydrates, free AAs, L-theanine, TP/AA, GA, caffeine, GC, C, EGCG, EC, CG, GCG, and ECG (Figure 5). In the biplot, the first principal component (F1) accounted for 52.49% of total variance and the second principal component (F2) accounted for 17.09% of total variance among four treatments tested in three seasons. F1 dimension was positively correlated with C, CG, EC, AA, GCG, carbohydrates, and L-theanine, but negatively correlated with EGCG, TP, GC, TP/AA, GA, ECG, and caffeine. The positive F2 dimension was largely correlated with L-theanine, C, GA, GC, caffeine, CG, EC, and ECG, while the negative F2 dimension was largely correlated with carbohydrates. The PCA results revealed tea leaf samples collected from fall were characterized with higher contents of carbohydrates, free AAs, L-theanine, and catechins including C, CG, EC, and GCG when compared with spring and summer, while tea leaves collected from spring and summer had the higher contents of EGCG, TP, GC, GA, caffeine, ECG, and high TP/AA. Tea leaf samples collected from no-shade control can be distinguished by a higher content of carbohydrates and lower content of L-theanine, GA, caffeine, and catechins among spring, summer, and fall when compared with black, blue, and red shade treatments.

## Discussion

Black and red shades increased PGIs of tea plants in 2018 and 2019 in this study, but there was no significant difference on PGIs between control and blue shade. Similar results were reported in other crops such as cabbage, snapdragon, marigold, and violet (12, 38, 39). Previous study showed that black shade nets absorbed all the ranges of light spectrum from 300 to 850 nm, blue shade nets had a high transmitting rate in the blue-green light spectrum range of 400 to 450 nm, while red shade nets had a high transmitting rate in orange-red light spectrum range of 590 to 850 nm (40). It was reported that red light and lower light intensities promoted stem elongation, while blue light inhibited stem elongation in peach, tomato, and cut flower plants (41–43). In a different report, red and blue light-emitting diodes were both reported to induce shoot elongation in 3-year-old tea plants (44). A newly transplanted tea field faces many challenges including weed pressure, drought, and heat stress. Fast growth and canopy expansion of tea plants can cover more field space to prevent light filtration to the ground and reduce the need for weed management (45). As a result, a higher or a faster-increasing PGI on tea plants under shade nets is beneficial for establishing a tea field.

In this study, plant growth indices of 2-year-old tea plants increased by 51, 49, 56, and 63% under no-shade control, black, blue, and red shades within one growing season from spring 2018 to spring 2019, respectively. Our previous study showed that 1-year-old tea plants of different cultivars increased their PGIs by 60–80% within a year in Mississippi (25). Growth rate of tea plants is affected by various factors such as cultivar, location, light condition, fertilization, and microorganism biodiversity in soil (46–49). The higher PGI under shades may result from the photosynthetic characteristics and microclimate under the shade nets. In this study, black, blue, and red shades showed similar effects in increasing *P*_n_ and *g*_s_, decreasing air and leaf temperatures, and reducing *PAR* to about 50% of full sun. Since air temperature was above 30°C when the photosynthesis data were collected, with *PAR* reaching 1597.73 μmol m^−2^ s^−1^, the unshaded tea plants (control) were exposed to high temperature and solar radiation stress, resulting in lower *P*_n_ and *g*_s_ compared with shaded tea plants. Similar results can be found in tomato plants grown in south Serbia, where *PAR* in open field in summer was reported to be approximately 1,600 μmol m^−2^ s^−1^, resulting in high light stress in unshaded tomato plants (50). Shade nets decreased air temperature by 2.08 to 2.19°C compared with no-shade control in this study, which agrees with previous studies showing that shading can decrease air temperature by 1–5°C (40, 51). *P*_n_ has been proven to be positively correlated with tea plant growth (52). The decreased air and leaf temperatures under shades can alleviate drought and heat stresses on tea plants in the long and hot summer in Mississippi.

According to recent study, there is an increasing frequency of frost due to the climate change, which is detrimental to tea production (53–55). In this study, we observed that black, blue, and red shades all alleviated frost damage on tea plants within our experiment duration. This result is consistent with reports showing that shade nets protected various crops, including apple and blueberry, from cold damage (56–58). Chilling temperature of 0–10°C starts to inhibit photosynthetic activities in leaves (59). Air temperature below freezing results in cellular damage expanding from leaf tip and margin to leaf center (60). Long period of freezing temperature would cause xylem embolism, causing stems and leaves to be desiccate after thawed (61, 62). Stem and leaf desiccation in tea plants was easily to find in February 2018 in the control group because of the freezing temperature in December 2017 and January 2018. Shade nets may protect plants by reducing radiation heat loss, increasing temperatures, and protect tea plants from radiation frost under shade in winter (63, 64). No difference in PGI, photosynthetic characteristics, and cold damage was detected among black, blue, and red shades, suggesting that spectral band may not affect tea plant growth significantly.

We found a wide range of variations on chemical components in tea leaves among different shade treatments and among the three harvesting seasons. Health benefits of tea in reducing risks of cardiovascular diseases, cancer, and obesity are highly related to the antioxidant activity of polyphenols (65). Catechins are the most abundant secondary metabolites in TP (66). Therefore, TP and catechins are important variables in the quality evaluation of tea. Our results agreed with previous reports in that shade treatments increased EGCG, the most predominant constituent in catechins compared with no-shade control (30, 66, 67). Total concentration of secondary metabolites as well as the secondary metabolites constituents (metabolite profile) in tea leaves is influenced by plant genotype, environment, cultivation method, etc. (68). TP/AA is a quality evaluation index for green tea, as lower TP/AA indicates higher quality green tea (69). Tea leaves collected from spring usually contain less TP and more AA, resulting in a lower TP/AA than in summer (65, 70–72). However, we detected higher TP and lower AA contents in spring than in summer among all the shade treatments, which likely resulted from the extreme low temperature in January 2018. A metabolomics study on green tea demonstrated that low temperature led to higher levels of EC, EGC, and EGCG (73). Similar results were found in central Africa, where the TP in tea leaves reached highest value in winter among seasons (74). The diversified seasonal L-theanine content in tea leaves was illustrated in different cultivars where the lower L-theanine content was found in spring than in fall or summer in some cultivars (25, 75). Further analysis of other individual AAs might will provide further explanation. When compared to no-shade control, black shade increased TP in all the three seasons, while blue and red shades increased TP in two seasons in this study. Similar result of shade decreasing TP in leaves was found on Scots pine in previous study (76). However, the result of TP differed from a previous report when 3 weeks of black shade treatment in spring reduced TP compared to no-shade control (30). In this study, the shade treatment was applied all year-round. Since shades decrease air temperature by decreasing solar radiation accepted by plants, the lower temperatures during winter months might lead to the higher TP under shade treatments. Short period of shade treatment may differ in its effects on temperature, RH, soil moisture, etc., compared with year-round shade treatments. Besides, tea plants used in this study were 2- and 3-year-old, which was younger than most tea plants in published study. Thus, further study on the comparison between short and long periods of shade treatment on mature tea plants is needed. Effects of various abiotic factors on secondary metabolite profile also merit further investigation.

Few previous studies analyzed the variation of carbohydrates in tea leaves among shade treatments or among seasons. Shade treatments generally reduced the carbohydrate content in tea leaves in this study, except that red shade resulted in the higher carbohydrate content than other treatments in summer. The highest content of carbohydrates was found in fall, which is consistent with our previous study (25). The carbohydrate results also helped us to separate samples collected from fall in PCA, with the highest contribution to the variations of 9.93% in F1. It has been reported that accumulation of carbohydrates in tea leaves may be related to plant adaptation to decreasing temperatures (77). AA is another major indicator for tea quality, which contributes brothy, sweet, and umami flavor to tea infusions (78). L-theanine is a unique nonproteinogenic AA, with health benefits such as reducing blood pressure, relieving stress, and anxiety (79, 80). The content of AA and L-theanine in tea leaves among all the treatments and harvesting seasons ranged from 3.20 to 3.77% and from 0.46 to 0.91%, respectively, within reasonable ranges as reported in previous studies (70, 81). Red shade increased the AA content compared to other treatments in spring and fall. All the shade nets increased L-theanine content in tea leaves among spring, summer, and fall, with red shade having more effect on L-theanine content than black and blue shades when compared with no-shade control. Accordingly, results in this study indicate that red shade may increase AA and L-theanine content in tea leaves, improving the quality of tea by enriching umami taste (82).

Gallic acid is one of the major phenolic acids in tea leaves (83). Caffeine is a central nervous system stimulant, which is widely consumed as an ingredient in food and beverages around the world (84, 85). In this study, contents of GA and caffeine ranged from 0.37 to 0.77% and 2.08 to 3.31%, similar to ranges reported by previous studies (65, 86). Deka et al. (65) reported that the highest content of caffeine in tea leaves harvested from July to August compared to April to May or October to November, consistent with seasonal trends in this study. Higher levels of caffeine synthase, which is an important enzyme in the caffeine synthesis of tea plants, were found in summer than in spring (87), which might explain our results at the metabolic level. Black, blue, and red shades increased the caffeine content in tea leaves among spring, summer, and fall, which is inconsistent with previous studies (32, 66, 67, 88). Although light is not an essential factor in the biosynthesis of caffeine, the higher levels of caffeine in tea leaves under shade treatments compared to no-shade control may result from the fact that light enhances the degradation of caffeine (89, 90).

## Conclusion

Use of black, blue, and red shades stimulated growth of 2- and 3-year-old tea plants grown in a subtropical climate in Mississippi, which may accelerate the establishment of a new tea field and alleviate weed pressure. Shade nets provided tea plants with protection from heat stress in summer and cold damage in winter and enhanced physiological responses including *P*_n_ and *g*_s_. The effects of black, blue, and red shades on tea plant growth were similar. Chemical compositions in tea leaves varied among shade treatments and harvesting seasons. In comparison with no-shade control, red shade increased the contents of L-theanine and free AAs in tea leaves in spring and fall, which are desirable characteristics for the green tea quality. Subsequent studies to investigate the effects of different color shades on the yield and quality of mature tea plants may provide valuable information for growers who are ready to produce tea.

## Data Availability Statement

The original contributions presented in the study are included in the article/Supplementary Material, further inquiries can be directed to the corresponding author.

## Author Contributions

GB, QZ, and JL designed and setup experiments. QZ, QW, and ZX carried out experiments. QZ analyzed experimental results. GB, TL, RH, and QW provided essential instruments and technical guidance. QZ wrote the manuscript that was revised by GB, TL, RH, QW, ZX, and JL. All authors contributed to the article and approved the submitted version.

## Funding

This study was funded in part by the USDA-Mississippi Department of Agriculture and Commerce Specialty Crop Block Grant Program (G00002402) and the USDA National Institute of Food and Agriculture Hatch Project MIS-249180.

## Conflict of Interest

The authors declare that the research was conducted in the absence of any commercial or financial relationships that could be construed as a potential conflict of interest.

## Publisher's Note

All claims expressed in this article are solely those of the authors and do not necessarily represent those of their affiliated organizations, or those of the publisher, the editors and the reviewers. Any product that may be evaluated in this article, or claim that may be made by its manufacturer, is not guaranteed or endorsed by the publisher.

## Supplementary Material

Supplementary Figure 1 High-performance liquid chromatography (HPLC) chromatogram of L-theanine in tea leaf sample.

Supplementary Figure 2 HPLC chromatogram of gallic acid (GA), caffeine, gallocatechin (GC), catechin (C), epigallocatechin-3-gallate (EGCG), epicatechin (EC), catechin-3-gallate (CG), gallocatechin-3-gallate (GCG), and epicatechin-3-gallate (ECG) in tea leaf sample.
