# Supplementary figures and images for: Color Shade Nets Affect Plant Growth and Seasonal Leaf Quality of Camellia sinensis Grown in Mississippi, the United States
Source: Front Nutr. 2022 Feb 2;9:786421. doi: 10.3389/fnut.2022.786421 (PMC8847693; doi:10.3389/fnut.2022.786421)

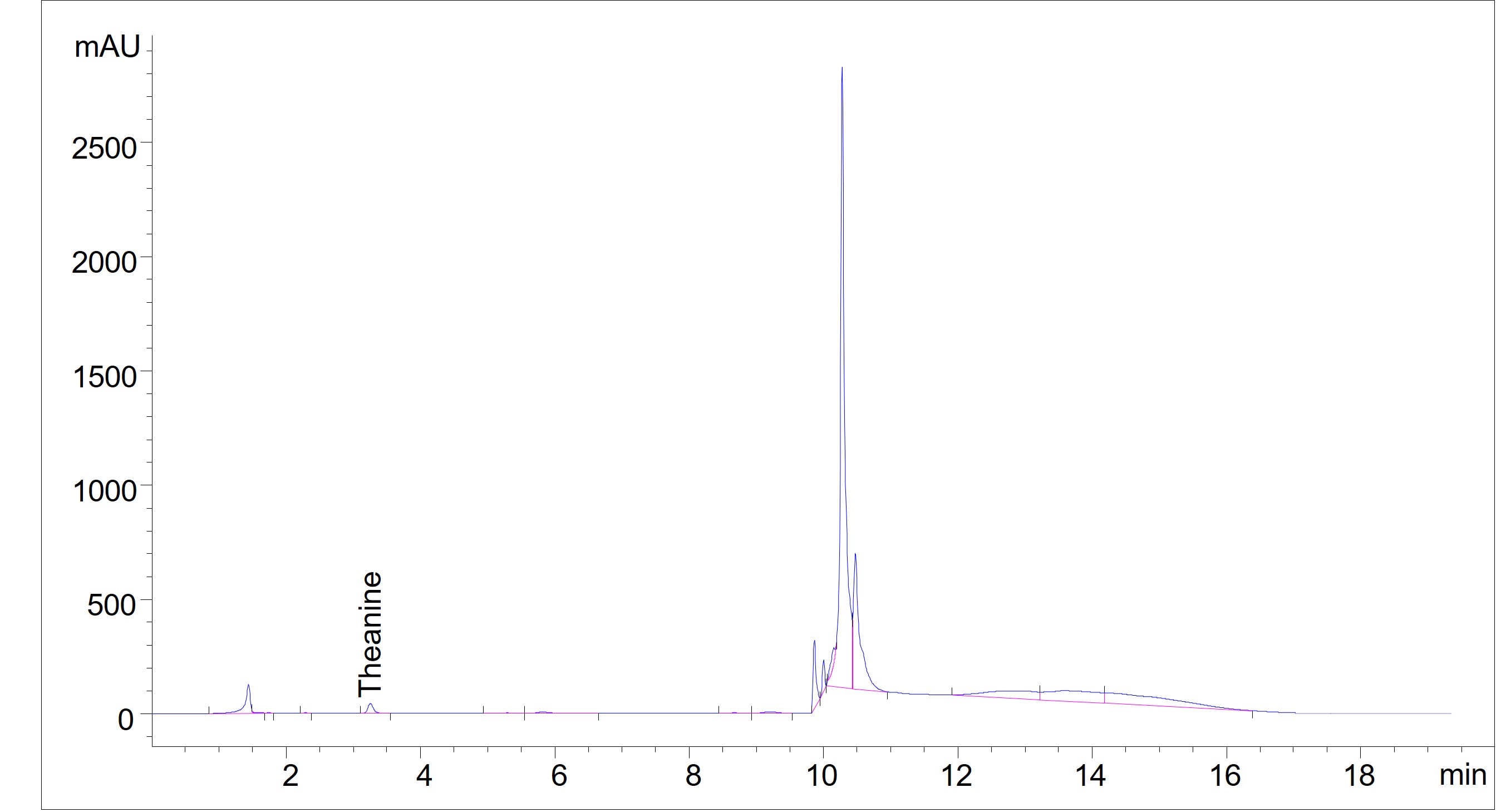

Supplement: Supplementary Figure 1 — High-performance liquid chromatography (HPLC) chromatogram of L-theanine in tea leaf sample. [file Image_1.JPEG]

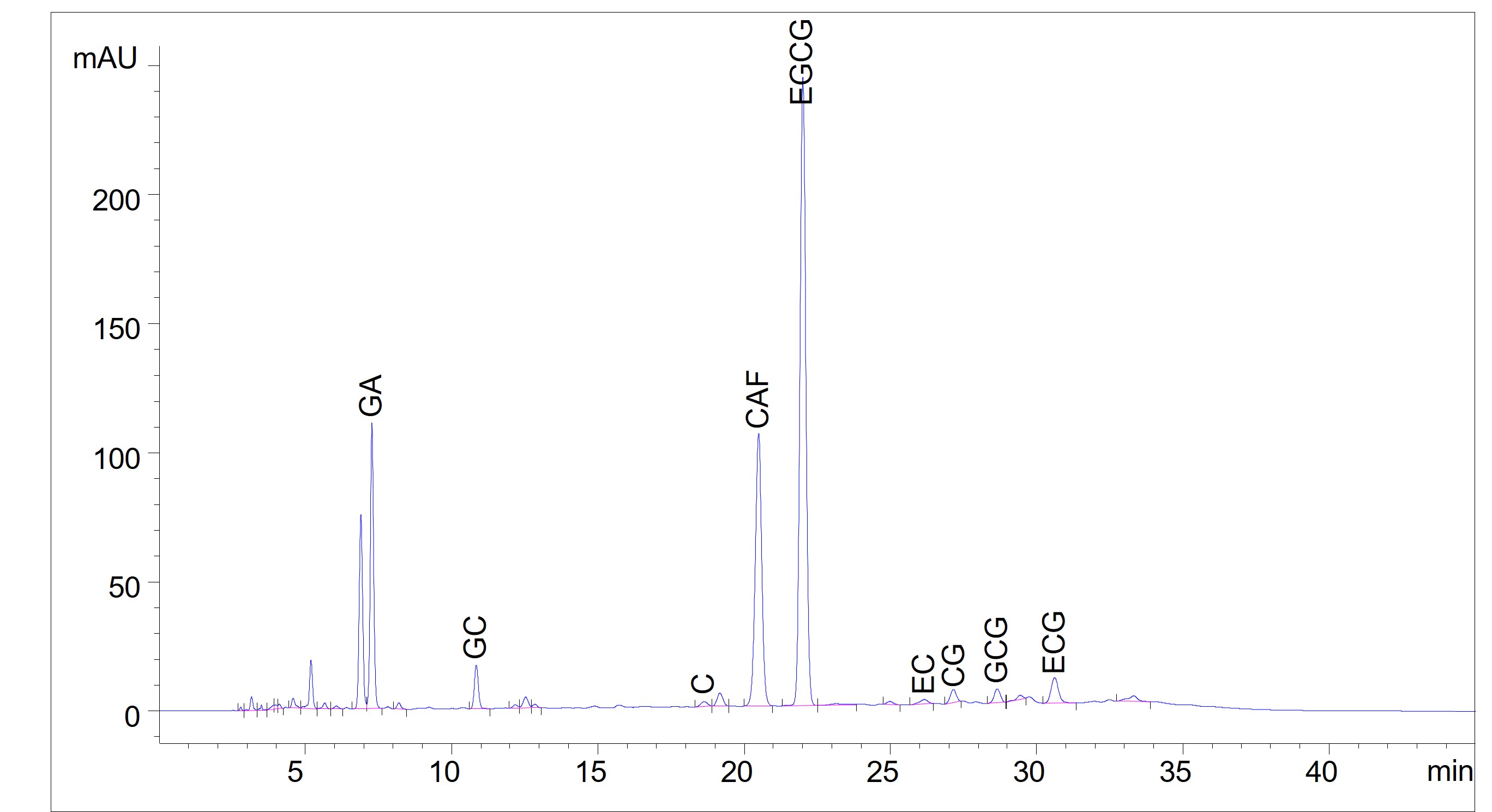

Supplement: Supplementary Figure 2 — HPLC chromatogram of gallic acid (GA), caffeine, gallocatechin (GC), catechin (C), epigallocatechin-3-gallate (EGCG), epicatechin (EC), catechin-3-gallate (CG), gallocatechin-3-gallate (GCG), and epicatechin-3-gallate (ECG) in tea leaf sample. [file Image_2.JPEG]
